# Dentin Abrasivity and Cleaning Efficacy of Novel/Alternative Toothpastes

**DOI:** 10.3290/j.ohpd.a45074

**Published:** 2020-09-04

**Authors:** Blend Hamza, Moritz Tanner, Thomas Attin, Florian J. Wegehaupt

**Affiliations:** a Resident, Clinic of Conservative and Preventive Dentistry, Center of Dental Medicine, University of Zurich, Zurich, Switzerland. Wrote the manuscript.; b Dental Master’s Student, Clinic of Conservative and Preventive Dentistry, Center of Dental Medicine, University of Zurich, Zurich, Switzerland. Performed the experiment in partial fulfilment for a Master’s degree.; c Professor and Clinic Director, Clinic of Conservative and Preventive Dentistry, Center of Dental Medicine, University of Zurich, Zurich, Switzerland. Performed critical evaluation of the experiment and the manuscript.; d Head of the Department of Preventive Dentistry and Oral Epidemiology, Center of Dental Medicine, University of Zurich, Zurich, Switzerland. Conceived and designed the experiment and critical evaluation of the manuscript.

**Keywords:** abrasive dentin wear, active carbon, alternative toothpastes, diamond powder, toothpaste abrasivity

## Abstract

**Purpose:**

To investigate dentin abrasivity and cleaning efficacy of novel/alternative toothpastes containing diamond particles, active carbon, sea salt or organic oils.

**Materials and Methods:**

Seventy-two bovine dentin samples (for measuring abrasivity) and 60 human dentin samples (for assessing cleaning efficacy) were used in this study. Samples were divided into six groups as follows: group 1: Elmex Kariesschutz (hydrated silica); group 2: Lavera Neutral Zahngel (sea salt); group 3: Curaprox Black is White (active carbon); group 4: Swiss Smile Diamond Glow (diamond powder); group 5: Ringana Fresh Tooth Oil (hydrated silica); and group 6: artificial saliva. Samples were brushed for a total of 26 min at 120 strokes/min, replacing slurries (1 part respective toothpaste and 2 parts artificial saliva) every 2 min. Finally, abrasive dentin wear was measured profilometrically and cleaning efficacy planimetrically.

**Results:**

The highest abrasivity values were observed for Lavera Neutral Zahngel (sea salt 9.2 µm) and Elmex Kariesschutz group (hydrated silica 6.0 µm). The lowest abrasivity value was observed for Ringana Fresh Tooth Oil group (hydrated silica 1.3 µm). The highest cleaning efficacy was observed for Elmex Kariesschutz group (86.7%) and the lowest cleaning efficacy was observed for Ringana Fresh Tooth Oil group (31.3%).

**Conclusion:**

The addition of diamond powder or active carbon to toothpastes could offer high cleaning efficacy with low dentin abrasivity. The addition of sea salt to traditional abrasives might cause high abrasive dentin wear without adding further cleaning benefit.

Daily toothbrushing with a fluoridated toothpaste is the most practiced oral hygiene habit for fighting dental plaque.^20^ However, fighting dental plaque is not the only reason that motivates people to practice daily oral hygiene. In a recent study investigating oral health knowledge and habits in three European countries, obtaining clean, white teeth was listed as an important motivation for daily toothbrushing.^8^

In order to remove dental plaque, toothpastes are loaded with abrasive particles.^4^ When dissolved in the toothpaste slurry, these abrasives should help remove dental plaque during brushing without harming the underlying tooth hard substance. However, clinical and laboratory observations have shown that a certain loss of hard tooth substance – especially dentin – can take place during toothbrushing, known as dental abrasion.^7^ Many factors play a role in the epidemiology of dental abrasion including the abrasivity of the toothpaste used, brushing frequency, and the force applied while brushing.^16^

Seeking alternative products which contain natural components and abstain from using specific chemical components has become a trend in the past few years. The oral-hygiene industry has responded to this trend and produced several alternatives to traditional toothpaste (eg, toothpastes with active carbon, organic oils, diamond particles, etc). However, the properties of such toothpastes have not yet been thoroughly investigated. It is therefore important that those toothpastes be tested for their qualities by independent researchers. This study was carried out to investigate the dentin abrasivity and cleaning efficacy of novel/alternative toothpastes. The null hypothesis was that there is no significant difference in the abrasivity and cleaning efficacy of the tested toothpastes.

## Materials and Methods

### Dentin Abrasivity

Seventy-two dentin samples were obtained from twelve bovine mandibular incisor roots. Under constant water cooling, six dentin samples were milled out from each root using a cylindrical diamond-coated trephine mill with an inner diameter of 3 mm. Each of the six samples from the same root was assigned to a different group, creating six groups of twelve dentin samples derived from twelve different roots. The samples were then embedded in acrylic resin (Paladur, Heraeus Kulzer; Hanau, Germany) using a silicon mold with an inner diameter of 6 mm. Placing the samples in the silicon mold ensured that only the outer part of the samples was circumferentially covered with acrylic resin. The acrylic resin was allowed to cure in a laboratory polymeriser (Palamat elite, Heraeus Kulzer) at 55°C and 2 bar for 10 min. The dentin surfaces were then ground and polished in an automatic grinding machine using 2000- and 4000-grit carborundum paper (waterproof silicon carbide paper, Tegramin-30, Struers; Copenhagen, Denmark) at 1 N pressure for 30 s and 40 s, respectively, under constant water cooling. This grinding removed the cement layer of the samples and resulted in a smooth dentin surface to facilitate subsequent profilometric determination of dentin abrasion.

In order to create a standard baseline situation, all samples were brushed for 500 cycles (1000 strokes) using a slurry of Elmex Kariesschutz toothpaste (Colgate-Palmolive; Swidnica, Poland). This preconditioning – and all following brushing sequences in this study – was carried out in a 6-place-cross-brushing-machine (custom-made, Clinic of Conservative and Preventive Dentistry, Center of Dental Medicine, University of Zurich, Zurich, Switzerland) using medium-hard standard toothbrushes (Paro M43, Esro; Thalwil, Switzerland). Every two samples were placed together in a brushing container which was screwed tight onto one of the six places of the brushing machine. The brushing frequency was set at 120 strokes/min and the load applied by the toothbrush on the samples at 1.6 N. After the preconditioning, all samples were rinsed thoroughly and stored in tap water until further use.

The groups were then brushed using six different toothpastes: group 1: Elmex Kariesschutz, positive control (abrasives: hydrated silica); group 2: Lavera Neutral Zahngel (Laverana; Wennigsen, Germany) (abrasives: silica, sea salt); group 3: Curaprox Black is White (Curaden; Kriens, Switzerland) (abrasives: hydrated silica, active carbon, nano-hydroxyapatite); group 4: Swiss Smile Diamond Glow (Curaden) (abrasives: hydrated silica, hydroxyapatite, diamond powder, silica); group 5: Ringana Fresh Tooth Oil (Ringana; Hartberg, Austria) (abrasives: hydrated silica, silica); group 6: artificial saliva,^11^ negative control. All tested toothpastes and their RDA values are shown in Table 1. Prior to the brushing sequence, baseline surface profiles of all samples were recorded with a stylus profilometer (Perthometer S2, Mahr; Göttingen, Germany). Five parallel surface profiles with a distance of 250 µm and a recording accuracy of 40 nm were recorded for each sample. The exact positioning and repositioning of the samples in the profilometer was ensured by using a prefabricated jig. Parts of the dentin samples with the adjacent acrylic resin were covered using a piece of adhesive tape, hence protecting the underlying areas from the brushing sequence and leaving about 1 mm of exposed dentin. These protected areas served later as reference areas for the profilometric analysis.

**Table 1:** Tested toothpastes, utilised abrasives incorporated, and RDA value according to the manufacturer

Toothpaste	Abrasive	RDA
Elmex Kariesschutz	Hydrated silica	65 (Tawakoli et al^18^)
Lavera Neutral Zahngel	Silica, Maris Sal (sea salt)	Not declared
Curaprox Black is White	Hydrated silica, carbon black (activated carbon), nano-hydroxyapatite	50 (declared by manufacturer)
Swiss Smile Diamond Glow	Hydrated silica, hydroxyapatite, diamond powder, silica	20 (declared by manufacturer)
Ringana Fresh Tooth Oil	Hydrated silica, silica	30 (e-mail correspondence with the manufacturer)

The slurries were prepared by mixing the respective toothpaste with artificial saliva at a ratio of 1:2 for 5 min. Two ml of the respective slurry was added to the brushing container with the samples of the respective group, then the brushing sequence was started. At 2-min intervals, the brushing sequence was stopped, the used slurry removed, 2 ml of fresh slurry was added to the brushing container, and the brushing sequence was resumed until the total 26-min brushing time was reached. After the brushing sequence, surface profiles (final profiles) were recorded again. Abrasive dentin wear was then determined with custom-made software which superimposed the baseline and final profiles. The profilometric analysis has been described in detail in a previous publication.^1^

### Cleaning Efficacy

For this part of the study, 60 human dental roots were separated from the crowns and cleaned of soft tissues using dental scalers. Teeth had been extracted anonymously in another clinic for reasons such as caries or periodontitis. Patients already consented – in written form – to the further use of their extracted teeth for research purposes and a written statement of the local ethics committee was obtained (BASEC Nr.:Req-2018-00867). Root surfaces were polished using light blue and light yellow Sof-Lex Pop-on disks (3M Oral Care; St Paul, MN, USA). Polishing was carried out under water cooling for 2 min for each disk and the load applied on the roots was set at 40-60 g using a pressure gauge. The samples were randomly divided into six groups (n = 10) and would later be brushed with the same above-mentioned toothpastes.

The samples were then stained in black tea for 17 h under gentle agitation and a constant temperature of 37°C. The tea was prepared by adding two different tea bags (Extra Strong, Marks & Spencer; Chester, UK, and Lipton Yellow Label, Unilever; Thayngen, Switzerland) to 390 ml of boiling water for 10 min. The pH value of the tea was set at 4 using citric acid.

**Fig 1:**
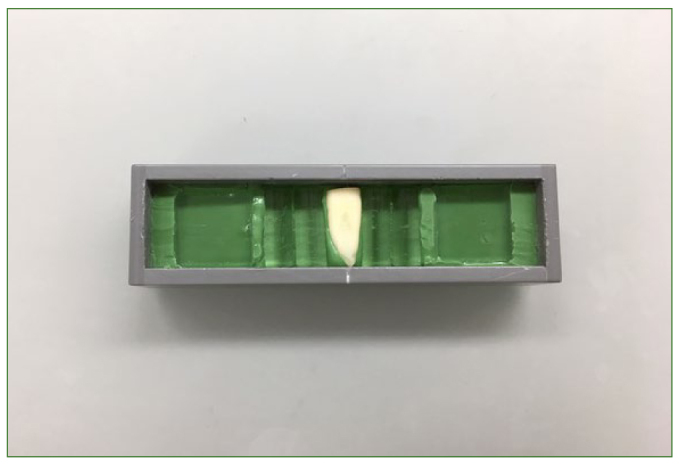
An embedded sample.

After staining, samples were rinsed with tap water and then embedded in a silicone material (President Plus light body, Coltène/Whaledent; Altstätten, Switzerland) inside a brushing container. Glass plates were also embedded on each side of the sample to ensure a smooth stroke movement of the toothbrush in the brushing container (Fig 1). The total to-be-brushed area was defined using the width of the toothbrush used and two parallel lines were accordingly engraved in the sample. Of each stained sample, a digital baseline image (Pentax K20D, Pentax; Wallisellen, Switzerland) was obtained in a dark room utilising a Tessovar (5X, Carl Zeiss; Feldbach, Switzerland).

Samples were then brushed with the respective slurry with same above-mentioned brushing protocol used to investigate the abrasivity. After 26 min total brushing time, samples were rinsed with tap water and new images were obtained using the same devices and standards as baseline. Pre- and post-brushing images of the same samples were compared with each other and visually analysed by the same investigator (MT). The total brushed area and the stain-free areas on the samples were outlined and analysed using Planimeter SC software (self-programmed; Clinic of Conservative and Preventive Dentistry, Center of Dental Medicine, University of Zurich, Zurich, Switzerland). The stain-free areas were expressed as a percentage of the total brushed area. The mean percentage obtained from all ten samples in one group was calculated and served as the value of the cleaning efficacy for the tested toothpaste. The experimental design is summarised in Table 2.

**Table 2:** Experimental design

Part 1: Dentin abrasivity					
72 dentin samples from 12 bovine roots					
Recording of baseline profiles					
Dividing into 6 groups. Samples in each group were derived from 12 different roots					
Group 1 n = 12	Group 2 n = 12	Group 3 n = 12	Group 4 n = 12	Group 5 n = 12	Group 6 n = 12
Brushing sequence (26 min; 1.6 N, 120 strokes/min, slurry 1:2, fresh slurry every 2 min)					
Elmex Kariesschutz	Lavera Neutral Zahngel	Curaprox Black is White	Swiss Smile Diamond Glow	Ringana Fresh Tooth Oil	Artificial saliva
Recording of final profiles					
Calculating the resulting abrasive dentin wear					
Part 2: Cleaning efficacy					
60 dentin samples from 60 human roots					
Random dividing into 6 groups					
Group 1 n = 10	Group 2 n = 10	Group 3 n = 10	Group 4 n = 10	Group 5 n = 10	Group 6 n = 10
Staining the samples with black tea modified with citric acid (17 h, 37°C, pH 4)					
Baseline digital images under Tessovar 5X					
Brushing sequence (26 min; 1.6 N, 120 strokes/min, slurry 1:2, fresh slurry every 2 min)					
Elmex Kariesschutz	Lavera Neutral Zahngel	Curaprox Black is White	Swiss Simle Diamond Glow	Ringana Fresh Tooth Oil	Artificial saliva
Digital images under Tessovar					
Analysing the stain-free surfaces planimetrically and calculate them as a percentage of the total brushing area					

### Statistical Analysis

The mean and standard deviation of the abrasive dentin wear in µm and cleaning efficacy in percentage were calculated for each group after 26 min. Differences between the groups were then tested using Wilcoxon signed-ranked tests (p < 0.05). All calculations were conducted using the statistical software R version 3.2.2 (The R Foundation for Statistical Computing; Vienna, Austria; www.R-project.org).

## Results

The resulting abrasive dentin wear varied considerably between the tested toothpastes in this study. Samples brushed with Lavera Neutral Zahngel showed the highest amount of abrasive dentin wear (sea salt 9.2 µm) followed by Elmex Kariesschutz (hydrated silica 6.0 µm), Curaprox Black is White (active carbon 3.0 µm), Swiss Smile Diamond Glow (diamond powder 1.9 µm), Ringana Fresh Tooth Oil (hydrated silica 1.3 µm) and artificial saliva (0.2 µm). All differences between the groups were statistically significantly different (p < 0.05).

**Fig 2:**
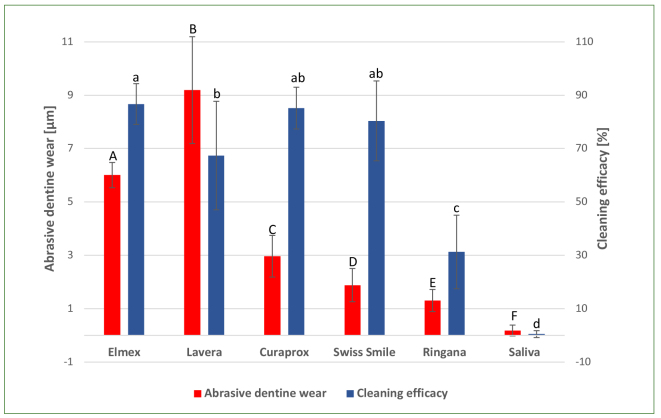
Mean values and standard deviation of abrasivity [µm] and cleaning efficacy [%] of the tested toothpastes. Values that are not statistically significantly different are marked with same letters (capital letters for abrasive dentin wear and lower-case letters for cleaning efficacy).

The highest cleaning efficacy was observed for Elmex Kariesschutz (hydrated silica 86.7%), Curaprox Black is White (Active carbon 85.2%) and Swiss Smile Diamond Glow (Diamond powder 80.4%) with no statistically significant difference between these groups (p > 0.05). The cleaning efficacy of Ringana Fresh Tooth Oil (hydrated silica 31.3%) was statistically significantly lower than that of Elmex Kariesschutz (hydrated silica), Curaprox Black is White (active carbon), Swiss Smile Diamond Glow (diamond powder) and Lavera Neutral Zahngel (sea salt 67.4%), but significantly higher than the cleaning efficacy of the artificial saliva (0.5 %). Figure 2 depicts both dentin abrasivity and cleaning efficacy of the tested toothpastes.

A cleaning:abrasivity ratio was calculated for the tested toothpastes by dividing the values for cleaning efficacy by the respective values for abrasive dentin wear.^24^ The higher the ratio, the better the cleaning efficacy and the lower the abrasivity offered by the respective toothpaste. The ranking of cleaning:abrasivity ratio for the tested toothpastes was as follows: Swiss Smile Diamond Glow (42.3), Curaprox Black is White (28.4), Ringana Fresh Tooth Oil (24.1), Elmex Kariesschutz (14.4), and Lavera Neutral Zahngel (7.3).

## Discussion

Obtaining clean, white teeth is an important impulse for people to brush their teeth.^8^ Toothpastes are loaded with abrasives to enhance the desired cleaning result during toothbrushing. However, abrasives can also cause a loss of the healthy tooth hard substance, i.e. abrasive dental wear.^10^ Moreover, toothpastes have become strongly associated with everyday life, which makes it important to keep track of their development and control their properties. This study was therefore carried out to investigate dentin abrasivity and cleaning efficacy of novel/alternative toothpastes on the market.

### Dentin Abrasivity

To measure toothpastes’ dentin abrasivity in this study, bovine teeth were used. Wegehaupt et al^22^ reported that bovine dentin is a suitable alternative to human dentin in abrasion studies. Because bovine teeth have larger surfaces, more samples can be derived from a single tooth and are more readily available than human teeth. Measuring abrasion by means of profilometric analysis has also been well described and utilised in the literature. In 1995, it was incorporated into the ISO specification (11609) for testing the abrasivity of toothpastes.^6^

All tested toothpastes in this study utilise silica or hydrated silica as abrasives. Hydrated silica comes in many different specifications not given in the INCI declaration. Hence, the fact that two toothpastes utilise the same abrasives does not necessarily mean that they have the same abrasivity value. Abrasive amount, shape, and agglomeration grade in each toothpaste play an important role in characterising its abrasivity.^4^ This could be applied to the different abrasivity values between Elmex Kariesschutz (6.0 µm) and Ringana Fresh Tooth Oil (1.3 µm). Since both toothpastes contain silica-based abrasives. Nevertheless, some of the tested toothpastes have non-classic or alternative abrasives (diamond powder, sea salt, hydroxyapatite, or activated carbon) along with the classic abrasives (silica or hydrated silica). In other words, the considerably higher abrasivity observed for the Lavera Neutral Zahngel group (sea salt 9.2 µm) or the lower abrasivity observed for Curaprox Black is White (active carbon 3.0 µm) or Swiss Smile Diamond Glow (diamond powder 1.9 µm) groups might also be attributed to the addition of the above-mentioned novel abrasives in addition to the potential differences in classic abrasives used in each of them.

### Dentin Abrasivity and Cleaning Efficacy

To assess the cleaning efficacy of the tested toothpastes, human dentin samples were stained with black tea, brushed with each toothpaste slurry, and planimetrically evaluated for cleaned areas. Black tea is consumed widely and known for its great ability to stain teeth. It has been used as the staining solution in several studies.^2,12^ Planimetric analysis has already been described and used to assess cleaning efficacy in several studies.^3,5,9,13,15,18,19^

The fact that samples were stained using a pH-4 black tea for 17 h leads to the logical assumption that they were simultaneously eroded. This duration is exaggerated and does not correspond to the quotidian situation in which teeth are in contact with staining agents for much less time. Moreover, it is safe to presume that the porous eroded dentin would absorb more staining solution than healthy dentin. The combination of these two factors (long staining time under erosive conditions) has created intense, clinically-unrealistic staining. Nevertheless, obtaining this intense staining was desired in this study to evaluate the performance of the tested toothpaste under such extreme conditions. It is also worth mentioning that some ingredients in the toothpaste might have a bleaching effect and could remove staining from the tooth surface without any mechanical aid. This effect could falsely be attributed to improved mechanical cleaning efficacy of the respective toothpaste. However, the intense staining of the samples in this study could have helped overcome this possible misleading bleaching effect.

As mentioned above, toothpastes need a certain amount of abrasives to fulfil their cleaning task. There is a general consensus in the literature that the abrasivity of toothpastes does not directly correlate with their cleaning efficacy.^14,18,23^ The results observed in this study seem to agree with this consensus. The cleaning efficacy presented by Elmex Kariesschutz (hydrated silica), Curaprox Black is White (active carbon) and Swiss Smile Diamond Glow (diamond powder) were comparably high. However, these three toothpastes had considerably different abrasivity values (see Fig 2). The fact that Curaprox Black is White (active carbon) and Swiss Smile Diamond Glow (diamond powder) produced the same cleaning efficacy but much less dentin wear than Elmex Kariesschutz (hydrated silica) raises the question of whether the higher abrasivity of Elmex Kariesschutz – or any other toothpaste for this matter – could be considered unnecessary. The same also applies for Lavera Neutral Zahngel, which caused the highest dentin wear but ranked fourth in cleaning efficacy. Taking this into consideration, and bearing in mind that cleaning is the main task of abrasives, the calculation of a cleaning:abrasivity ratio for toothpastes seems helpful to determine whether the abrasivity of a toothpaste is justified. However, the best method to be used to assess cleaning efficacy and abrasivity is still a matter of debate.

The fact that a toothpaste containing sea salt caused more abrasive dentin wear than toothpastes containing much harder active carbon or diamond powder is interesting. Due to its solubility in water, the abrasivity of sea salt should be rather low; thus, the high abrasivity of Lavera Neutral Zahngel is more likely attributable to the presence of silica. However, the co-presence of the alternative abrasives might mask or synergise the abrasive dentin wear caused by traditional abrasives. It should be emphasised that toothpastes in this study were neither tested for their abrasivity towards enamel nor for their surface roughness values. Both of these properties are important to gain a better understanding of how novel/alternative toothpastes interact with teeth. Wegehaupt et al^21^ found a toothpaste with diamond powder to be highly abrasive on enamel compared to toothpastes with traditional abrasives. This large difference in abrasivity behaviour of diamond powder on enamel and dentin is interesting. The mild abrasivity of diamond powder on dentin could be attributed to the fact that diamond is hard enough to push the dentin surface inward rather than to become abraded when rubbed by diamond particles. Other studies have also shown diamond-containing toothpastes to possess low abrasivity on dentin.^17^

The abrasivity and cleaning efficacy of Ringana Fresh Tooth Oil was rather low. It is improbable that oil has a direct abrasive effect. However, the gliding effect of oil could alter the way classical abrasives interact with the tooth surfaces and thus reduce the abrasive dentin wear that they could have otherwise caused.

The null hypothesis of this study must be rejected as the tested toothpastes exhibit significantly different abrasivity and cleaning efficacy values.

## Conclusion

Within the limits of this study, it could be concluded that the addition of alternative/novel abrasives, namely diamond powder and active carbon, to traditional abrasives could enhance the resulting cleaning efficacy while keeping abrasive dentin wear low.
